# Young Hispanics at risk of type 2 diabetes display endothelial activation, subclinical inflammation and alterations of coagulation and fibrinolysis

**DOI:** 10.1186/1758-5996-5-37

**Published:** 2013-07-19

**Authors:** Carlos O Mendivil, Ludivina Robles-Osorio, Edward S Horton, Osama Hamdy, Augusto Enrique Caballero

**Affiliations:** 1 Universidad de los Andes Medical School, Bogotá, Colombia; 2 Joslin Diabetes Center, Clinical Research Center, Harvard Medical School, Boston, MA 02115, USA

**Keywords:** Diabetes, Cardiovacular disease, Endothelial, ICAM-1, Hispanics, Prevention

## Abstract

**Background:**

Hispanics have a high rate of diabetes that exposes them to an increased risk of cardiovascular disease. We hypothesized that many of the pathophysiological mechanisms that cause atherosclerotic disease may be present in young Hispanics who do not have clinical diabetes but are at increased risk of developing it.

**Methods:**

We studied 36 young Hispanic adults without diabetes (ages 18–40). Seventeen participants were at increased risk of developing type 2 diabetes given by overweight and a family history of diabetes on one or both parents (at risk group). Nineteen participants with normal body-mass index and no parental history of diabetes constituted the control group. We measured and compared plasma markers of endothelial dysfunction, disturbed coagulation and fibrinolysis, subclinical inflammation and adipose tissue dysfunction in the at risk and control groups.

**Results:**

Participants at risk of diabetes were more insulin-resistant according to different indicators, and had significantly higher levels of soluble intercellular adhesion molecule-1 (sICAM-1), tissue plasminogen activator (tPA), inhibitor of plasminogen activator-1 (PAi-1), high sensitivity C-reactive protein and free fatty acids, signaling the presence of multiple proatherogenic alterations despite the absence of overt diabetes. Levels of the prothrombotic molecule PAi-1 were most elevated in participants who were not only at risk of diabetes by the study definition, but also abdominally obese.

**Conclusions:**

Young adult Hispanics at risk of type 2 diabetes but without overt disease already bear considerably high levels of markers reflecting processes that lead to the development of atherosclerotic cardiovascular disease.

## Background

People of Hispanic or Latino origin are currently the largest and fastest-growing minority group in the United States [1]. Among Hispanics in the United States, the prevalence of type 2 diabetes mellitus has been increasing at an astonishingly fast rate during the last three decades. Between the years 1980 and 2006, this prevalence jumped from 2.6% to 10.4% [2], an increase equally marked in all Hispanic subgroups [2]. Type 2 diabetes is a strong cardiovascular disease (CVD) risk factor [3], and the dramatic rise in its prevalence that we are witnessing today is likely to translate into increased cardiovascular morbidity, mortality and health costs for the Hispanic population in the future. This would add to the already significant burden of CVD in the Hispanic population. Not only is CVD the main cause of death among Hispanics [4], but when the prevalence of coronary artery calcification is estimated by ethnicity, Hispanics have the second highest prevalence after whites [5]. Furthermore, Hispanic immigrants in the US have a particularly high prevalence of overweight or obesity, another relevant cardiovascular risk factor [6]. Importantly, Hispanics tend to deposit visceral and subcutaneous abdominal fat as young adults rather than later in life, so many of the metabolic derangements contributing to cardiovascular risk in this population may be present decades before CVD is manifest [7].

Throughout the atherothrombotic process that ends in a clinical CVD event, endothelial dysfunction, hypercoagulability and subclinical inflammation have proven to be key pathophysiologic factors [8], and a number of biomarkers that measure these alterations have been discovered and developed [9]. Biomarkers that measure endothelial dysfunction or subclinical inflammation are also predictive of the risk of developing type 2 diabetes [10,11], so they may provide very useful information for opportune preventive interventions aimed at both type 2 diabetes and CVD.

Alterations in endothelial homeostasis are present in individuals who bear risk factors for the development of type 2 diabetes well before the development of clinical hyperglycemia [12,13], and prior studies have encountered high levels of markers of endothelial dysfunction, coagulation/fibrinolysis alterations and subclinical inflammation in Caucasian patients with obesity [14], or with a family history or a personal diagnosis of diabetes [15]. A widely held belief is that atherosclerotic disease is a problem of middle-aged and older adults, and despite some data on Hispanic youth [16], very little is being done to explore the alterations that mediate the high burden of CVD among Hispanics, and open opportunities for early intervention focusing on relevant factors.

For this reason, our main study aim was to explore whether plasma markers of endothelial dysfunction (soluble intercellular adhesion molecule-1 [sICAM-1] and soluble vascular adhesion molecule [sVCAM]), disturbed coagulation/fibrinolysis (tissue-type plaminogen activator [tPA] and plasminogen activator inhibitor-1 [PAi-1]), subclinical inflammation (high sensitivity C-reactive protein [hsCRP] and interleukin 6 [IL-6]) are increased, while markers of adipose tissue function (adiponectin) are reduced; in young Hispanic adults who do not have clinical type 2 diabetes but are at increased risk of type 2 diabetes because of their body weight and family history.

### Research design and methods

The protocol was approved by the Committee on Human Studies of the Joslin Diabetes Center (JDC) and the Scientific Advisory Committee of the Clinical Research Center at the Beth Israel Deaconess Medical Center. All research activities were conducted according to the principles expressed in the declaration of Helsinki. All participants provided written informed consent.

The study included young Hispanic adults at risk of developing type 2 diabetes (“at risk” group), and subjects of the same age group and ethnic background but without the selected risk factors for type 2 diabetes (“control” group). All subjects were Hispanic, between 18 and 40 years old, and had not participated in any exercise program for the 6 months prior to the start of the study.

We considered a subject Hispanic if both parents reported to be Hispanic when asked the following question: “What ethnic group do you consider that you belong to?”. For the at risk group, subjects had to have a history of type 2 diabetes in one or both parents, and a body-mass index (BMI) above 27 Kg/m^2^. For the control group, subjects had to have no history of type 2 diabetes in any first degree relative, and a BMI under 25 Kg/m^2^. At-risk and control subjects were matched by age in +/− 5-year brackets, that is, a participant in the “at-risk” group who was 30 years old was matched to a control subject of the same sex, with an age between 25 and 35 years. The exclusion criteria included diagnosed diabetes or treatment with oral antidiabetic drugs, insulin or lipid-lowering drugs, pregnancy, smoking, existent cardiovascular disease, uncontrolled arterial hypertension, renal disease, proteinuria, cancer, infectious diseases, severe GI diseases, lung diseases, electrolyte abnormalities, anemia, peripheral vascular disease, use of beta-blockers, any diuretic, calcium channel blockers, ACE inhibitors, angiotensin receptor blockers, niacin, glucocorticoids, any antineoplastic agent, antibiotics, psychoactive agents or bronchodilators.

Study subjects were recruited from the offspring of patients with type 2 diabetes at the Joslin Diabetes Center and the Beth Israel Deaconess Medical Center in Boston, notices placed on bulletin boards at the medical center and in diabetes clinics, or by referral from colleagues. Only one subject from any given family was included in the study.

For the study protocol, patients arrived at the clinical research center of the JDC after a 12 hour fast, had a full medical history and physical examination, plus measurement of weight, height, and waist and hip circumferences by nutrition staff to ensure consistency. Blood samples were taken for the measurement of plasma markers and general chemistry. An oral glucose tolerance test (OGTT) was performed in accordance with World Health Organization recommendations. The glucose load was calculated as 1.75 grams of glucose/Kg, with a maximum amount of 75 g. Samples for plasma glucose and insulin were collected at baseline and 30, 60, 90, and 120 min after the oral glucose load.

In the fasting blood samples, we measured glucose, insulin, glycated hemoglobin A1c (A1c), lipids, free fatty acids, urea nitrogen, creatinine, sVCAM, sICAM-1, PAi-1, tPA, TNF-alpha, IL-6, hsCRP and adiponectin.

Plasma glucose, total serum cholesterol, triglycerides, blood urea nitrogen, and creatinine were measured using the Synchron CX analyzer (Beckman Systems, Oxford, CT), whereas HDL cholesterol was measured directly (Polymedco, Cortland Manor, NY). LDL cholesterol concentration was estimated by the Friedewald formula [17] or was directly measured (Polymedco) if triglycerides were > 250 mg/dl, total cholesterol was >240 mg/dL, or HDL cholesterol was <35 mg/dL. A1c was determined using ion-exchange, high-performance liquid chromatography. Insulin (Immulite, two-site chemiluminescent immunometric assay; Diagnostic Products, Los Angeles, CA), free fatty acids (enzymatic colorimetric method, NEFA C kit; Waco Chemicals, Richmond, VA), and all vascular markers and adipocytokines were measured at the core laboratory of the clinical research center at the Beth Israel Deaconess Medical Center, Boston, Massachusetts. Accepted commercial kits used were sICAM-1 and sVCAM (ELISA; R&D Systems, Minneapolis, MN), TNF-alpha and IL-6 (ELISA; R&D Systems), hsCRP (solid-phase, chemiluminescent immunometric assay; Diagnostic Products), PAi-1 and tPA (ELISA; Diagnostica Stago, Parsipanny, NJ), and adiponectin (ELISA; Linco Research, St. Charles, MI). We evaluated the glucose and insulin area under the curve (AUC) with the trapezoidal method: ½ Σ (t_i+1_ - t_i_) (y_i_ + y_i+1_) [18].

Common indexes of insulin sensitivity and insulin secretion were used: homeostasis model assessment (HOMA) of insulin resistance [19] (HOMA-IR: {fasting insulin [microU/mL] * fasting glucose [mmol/L]}/22.5), HOMA of beta-cell function (HOMA beta-cell% = [20 * fasting insulin]/[glucose (mmol/L) - 3.5]), insulin sensitivity index (ISI: 22.5/(fasting insulin [microU/mL]*fasting glucose [mmol/L]), and the 1/fasting insulin index. We used insulin secretion indexes as the corrected insulin response (CIR: 100 * 30 min insulin/[30 min glucose (mg/dL) * {30 min glucose (mg/dL) - 70 mg/dL}]) and the insulin-to-glucose ratio: (30 min insulin [microU/mL] - fasting insulin [microU/mL])/(30 min glucose [mg/dL] - fasting glucose [mg/dL]) (19). Total body and trunk fat were estimated through dual energy X-ray absortiometry with a Hologic QDR 4500 densitometer machine (Hologic, Bedford, MA).

### Statistical analysis

The sample size was calculated to achieve 80% statistical power at a two-sided significance level of 0.05 for a *t* test in order to identify a difference in the plasma markers of endothelial function (sICAM-1) of at least 6% between the groups, based on previous data on overweight adults [14]. An expected difference between the groups of 30 ng/ml for sICAM, with an expected SD of 27 ng/mL, would require at least 14 subjects in each of the groups. Statistical analyses were performed using SPSS for Windows, version 17 (SPSS, Chicago, IL). For all comparative data, an alpha-error rate of 0.05 and a beta-error rate of 0.2 were considered. The Kolmogorov-Smirnov test was used to assess the normality assumption of continuous distribution. The results are presented as means +/− SD, median, 25th-75th percentile intervals (interquartile range), and percentages, as applicable. For normally distributed continuous variables, two-sided *t* tests were used for the comparison of differences between groups. Non-normally distributed continuous variables were compared using the Wilcoxon signed-rank test. All categorical variables were analyzed using the chi-square test or Fisher’s exact test when applicable. Spearman correlations were performed to evaluate the association of continuous variables. To explore the influence of central body fat distribution, we classified study participants by waist circumference above or below the respective group median, and compared mean levels of markers using a two-way ANOVA with level of waist circumference and risk group as fixed factors.

## Results

Thirty-eight subjects were screened for the study, and 2 were excluded due to a diagnosis of diabetes on the oral glucose tolerance test (OGTT), for a final sample size of 36 subjects: 19 in the control group and 17 in the at risk group. There were 9 women in the control group and 10 women in the at risk group. As discovered in the OGTT, 5 participants in the at risk group had impaired glucose tolerance (IGT) (29.4% of the group), and 2 had impaired fasting glucose (IFG) (11.8% of the group). No participant in the control group had IFG or IGT.

Participants in the at risk group had by study design a higher weight and BMI than the control group (Table 1). They also showed higher mean values of several measures of body adiposity, but not higher A1c levels. Concerning plasma lipids, the only significant differences were higher triglycerides and free fatty acids in the at risk group.

**Table 1:** Demographic and clinical characteristics of the two study groups

	**Risk group**		**P**
	**Control**	**At risk**	
N=	19	17	-
Age (years)	28.3 ± 5.6	31.8 ± 5.5	0.07
Female sex (%)	47.3	58.8	0.59
Weight (Kg)	65.4 ± 12.1	87.5 ± 21.0	<0.001
Body mass Index (Kg/m^2^)	23.0 ± 2.0	32.2 ± 5.9	<0.001
Waist circumference (cm)	78.9 ± 7.5	99.1 ± 14.8	<0.001
Waist-to-hip ratio	0.82 ± 0.06	0.87 ± 0.07	0.014
Whole body fat (%)	24.2 ± 6.4	35.9 ± 7.1	<0.001
Trunk body fat (%)	23.0 ± 5.8	36.7 ± 7.8	<0.001
Systolic blood pressure (mmHg)	105.4 ± 13.7	112.4 ± 9.6	0.09
Diastolic blood pressure (mmHg)	67.9 ± 7.5	74.7 ± 9.7	0.025
Fasting plasma glucose (mmol/L)	4.70 ± 0.32	5.25 ± 0.43	<0.001
OGTT 2 h plasma glucose (mmol/L)	7.03 ± 2.16	8.23 ± 2.74	0.14
Impaired fasting glucose # (%)	0 (0)	2 (11.8)	0.12
Impaired glucose tolerance # (%)	0 (0)	5 (29.4)	0.011
Glycated hemoglobin A1c (%)	5.2 ± 0.2	5.2 ± 0.7	0.6
Total cholesterol (mmol/L)	4.45 ± 0.78	4.34 ± 1.04	0.74
Triglycerides (mmol/L)	0.84 ± 0.45	1.26 ± 0.64	0.026
HDL cholesterol (mmol/L)	1.23 ± 0.40	1.01 ± 0.25	0.067
LDL cholesterol (mmol/L)	2.87 ± 0.71	2.91 ± 1.06	0.91
Triglycerides/HDL cholesterol ratio	1.78 ± 1.2	3.15 ± 1.91	0.015
Plasma free fatty acids (mEq/L)	0.42 ± 0.16	0.58 ± 0.22	0.02

Values are means (SD) unless indicated otherwise.

### Measures of glucose homeostasis and insulin sensitivity

**Figure 1:**
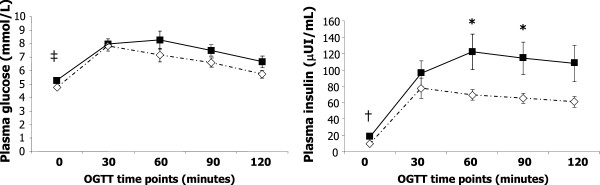
**Plasma glucose and insulin concentrations during the OGTT in the control (light diamonds) and obese (dark squares) groups.** *P < 0.05, †P < 0.01, ‡P < 0.001.

Fasting plasma glucose levels were significantly higher in the group at risk (5.25 +/− 0.43 mmol/L) than in the control group (4.7 +/− 0.32 mmol/L) (p < 0.001). However, there were no significant differences on plasma glucose levels between groups at subsequent time points of the OGTT (Figure 1). Insulin levels during the OGTT were in general higher and more variable in the group at risk. The difference in fasting plasma insulin was significant (18.0 +/− 2.3 μUI/mL in the group at risk and 9.8 +/− 5.0 μUI/mL in the control group, p = 0.018), and insulin levels at time points 60 min and 90 min during the OGTT were also significantly higher in the group at risk. The between-groups difference in insulin at 120 min was of borderline statistical significance (p = 0.058).

There was a large and significant (p = 0.02) between-groups difference in the insulin AUC, with the average value being almost twice as high in the group at risk as in the control group (Table 2). Glucose AUC was also significantly higher in the group at risk, although the proportional difference between groups was smaller. Participants in the group at risk also had significantly higher values of the HOMA-IR index, and a lower Insulin Sensitivity Index of borderline statistical significance (p = 0.051). Between-groups differences in other measures of insulin resistance were not statistically significant.

**Table 2:** Measures of glucose homeostasis and insulin sensitivity in the two study groups

	**Risk group**		**P**
	**Control**	**At risk**	
Insulin AUC (microUI*min^-1^*mL^-1^)	7450 ± 3156.4	11998.1 ± 7343.9	0.020
Glucose AUC (mg*min^-1^*dL^-1^)	14505.8 ± 2718.3	16718.7 ± 3766.8	0.042
Corrected Insulin Response	0.56 ± 1.37	1.09 ± 0.91	0.238
Insulin-to-glucose ratio	1.28 ± 1.45	2.14 ± 1.94	0.129
Insulin Sensitivity Index	0.6 ± 0.27	0.38 ± 0.4	0.051
HOMA-IR	2.11 ± 1.17	4.35 ± 2.79	0.004
HOMA-beta cell	177.4 ± 167.8	203.3 ± 127.6	0.299
1/Insulin	0.13 ± 0.06	0.09 ± 0.08	0.073

### Markers of endothelial function and subclinical inflammation

**Figure 2:**
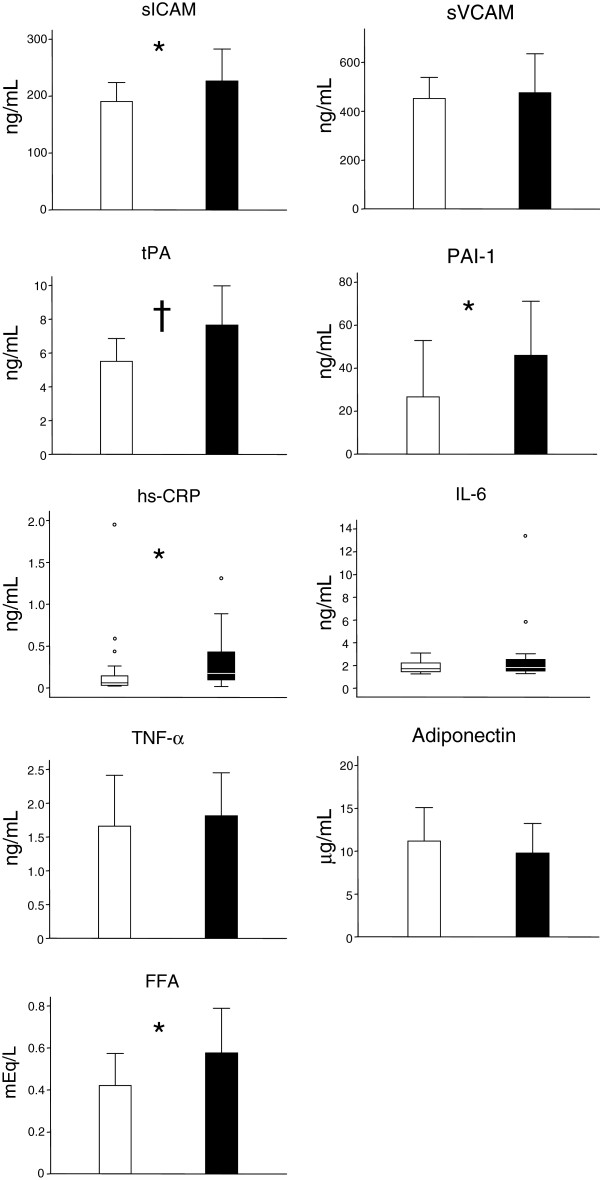
**Markers of endothelial activation, coagulation and fibrinolysis, vascular inflammation, and adipocytokines in the control (light bars) and at risk (dark bars) groups.** The variables with a normal distribution are shown with bars and SDs, whereas those not normally distributed are shown in a box plot format. *P < 0.05, †P < 0.01, ‡P < 0.001.

The group at risk had increased levels of sICAM-1, but not sVCAM; as well as significantly elevated tPA, PAi-1, hsCRP and free fatty acids. Adiponectin levels were on average lower in the group at risk, but this difference was not significant (p = 0.3). The most pronounced between-groups difference was evident for the coagulation/fibrinolysis markers (Figure 2). No difference was observed on circulating TNF-alpha levels.

### Relationship between fat distribution and biomarkers

**Figure 3:**
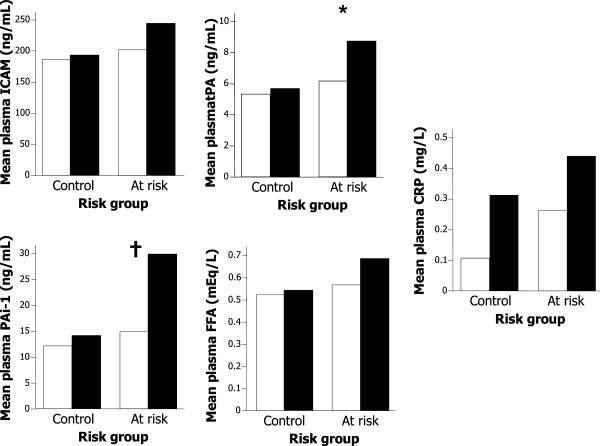
**Markers of endothelial activation, coagulation, fibrinolysis and vascular inflammation that differed between the control and at risk groups, by waist circumference below (light bars) or above (dark bars) the median of their risk group.** Median waist circumference was 94 cm in the at risk group, and 80.5 cm in the control group. *p = 0.02 and †p = 0.04 for difference between high and low waist circumference in a two-way ANOVA that included risk group.

Among participants in the at risk group, those with a waist circumference above the group median had significantly higher levels of PAi-1 compared to those with waist circumferences below the median (Figure 3). Participants in the at risk group with waist circumferences below the group median had PAi-1 levels comparable to those of participants in the control group. Concentrations of hsCRP were on average higher among participants with waist circumferences above their group median, but this difference did not reach statistical significance in either group.

### Correlations between glucose homeostasis indexes and biomarkers

The highest correlations were observed between PAi-1 or tPA and the measures of insulin resistance: fasting insulinemia, insulin AUC and HOMA-IR, Spearman correlation coefficients ranged between 0.67 and 0.71 (p < 0.001 for all correlations). There was also a positive and significant correlation of PAi-1 (r = 0.53, 0.001) and tPA (r = 0.65, p < 0.001) with fasting glucose. sICAM-1 levels were most strongly correlated with the insulin AUC (r = 0.61, p < 0.001), but also showed significant correlations with fasting glucose (r = 0.41, p = 0.011), fasting insulin (r = 0.52, p < 0.001), HOMA-IR (r = 0.54, p < 0.001) and HOMA-beta cell (r = 0.37, p = 0.026). Meanwhile, sVCAM did not correlate with any of the performed indexes of glucose metabolism. Among the biomarkers of subclinical inflammation, hsCRP was the one most strongly correlated with metabolic variables including fasting glucose (r = 0.50, p = 0.002), fasting insulin (r = 0.50, p = 0.001), insulin AUC (r = 0.48, p = 0.002), HOMA-IR (r = 0.53, p < 0.001) and HOMA-beta cell (r = 0.34, p = 0.038). On the other hand, IL-6 was not significantly associated with any metabolic variable, and circulating TNF-alpha levels only correlated with insulin AUC (r = 0.34, p = 0.045). Adiponectin was significantly and negatively correlated with all measures of insulin resistance, coefficients were r = −0.43 (p = 0.01) with fasting glucose, r = −0.46 (p = 0.004) with fasting insulin, r = −0.34 (p = 0.04) with insulin AUC and r = −0.51 (p = 0.001) with HOMA-IR. Plasma free fatty acids correlated positively with the glucose AUC (r = 0.48, p = 0.003).

## Discussion

In this study in young Hispanic adults without diabetes or other metabolic disease, an increased risk for type 2 diabetes was significantly associated with markers of early phenomena in the pathogenesis of atherosclerotic disease: Endothelial activation (higher levels of sICAM-1), subclinical inflammation (higher levels of hsCRP), disturbed coagulation/thrombolysis (increased levels of PAi-1 and tPA), and impaired suppression of adipose tissue lipolysis (increased levels of plasma free fatty acids). The interpretation of our central finding could be that in young Hispanic adults without any overt metabolic disease but at risk of type 2 diabetes, the pathophysiological alterations that lead to macrovascular disease were already present. The average age of both the control and the at risk group was around 30 years, so they do represent young Hispanic adults, despite the relatively wide range of ages allowed in the inclusion criteria. This supports the idea that preventive measures aimed at preventing the silent progression of cardiovascular disease should be instated since the early adulthood, particularly among ethnicities disproportionately affected by diabetes and cardiovascular disease, as is the case among Hispanics.

sICAM-1 is a circulating version of the adhesion molecule Intercellular Adhesion Molecule-1 (ICAM-1), which facilitates adhesion of circulating monocytes to endothelial cells [20]. sICAM is not an entirely endothelium-specific marker and its levels may increase during acute inflammatory processes but it has the great advantage that, being a circulating marker; it allows for a global evaluation of endothelial function; as opposed to the local assessment of endothelial function by the measurement of endothelium-dependent vascular reactivity. In prospective studies, sICAM-1 has been positively associated with the risk of myocardial infarction among initially healthy individuals [21,22]. There seem to be differences in the prognostic meaning of sICAM-1 and sVCAM: a previous observational study found a positive association between sICAM-1, but not sVCAM and atherosclerotic disease, in that case carotid or femoral plaque burden [23]. The same observation of sICAM-1 being a better predictor was found in a large cohort study of endothelial markers and symptomatic peripheral arterial disease [24]. This evidence suggests that the elevated sICAM-1 of individuals in the at risk group in our study signals an increased risk of future CVD despite their apparently healthy condition.

tPA is a serine protease that degrades fibrin, promoting the dissolution of pre-formed blood clots. PAi-1 is a potent inhibitor of tPA, what makes it an essentially prothrombotic mediator, but it also has important profibrotic and chemotactic activities [25]; both tPA and PAi-1 have been reported to correlate with body weight [26]. The very large between-groups difference in our study (levels in the at risk group almost doubled those in the control group); emphasizes that apparently healthy young individual at risk for type 2 diabetes exhibit remarkable alterations in these markers. An appealing finding was that PAi-1 levels in the at risk group were elevated only among participants with a waist circumference above the group median (a relative measure of abdominal obesity). In other words, the association between being at risk for type 2 diabetes and PAi-1 levels was mostly accounted for by abdominal obesity. Prior studies have documented a strong association between abdominal adiposity and circulating PAi-1 in subjects without diabetes [27,28]. It has also been shown in animal models that insulin resistance, which is strongly associated with abdominal obesity; stimulates the production of PAi-1 through the Mitogen-Activated Kinase pathway [29]. This suggests that interventions focused on reduction of adiposity and improvement of body fat distribution may have an important positive impact on coagulation disturbances among Hispanics at risk for type 2 diabetes.

The elevated hsCRP levels of the at risk group confirm that subclinical inflammation is a key precursor of the metabolic alterations that foster the appearance of type 2 diabetes. There is a well established association between hsCRP and body adiposity, hence part of the between-groups difference in hsCRP may be attributed to BMI differences; but it was interesting to observe that higher waist circumferences were associated with higher hsCRP levels even within the control group. The hsCRP difference between control subjects above or below the waist circumference group median did not achieve statistical significance, but there was a clear trend towards increased hsCRP in the relatively more abdominally obese control subjects. The lack of significance for such a large difference is probably due to limited sample size and the intrinsically high biological variability of hsCRP. In any case, the significantly higher hsCRP levels in the at risk group constitute a worrying alarm, given that hsCRP correlates strongly with the long term risk of type 2 diabetes, CVD, stroke and death [30,31]. The other two inflammatory markers besides hsCRP (IL-6 and TNF-alpha) were not associated with at risk status, there may be several reasons for this. IL-6 is known to stimulate production of hsCRP by the liver, so in a sense hsCRP is an integrated version of IL-6 activity, one that may be more related to chronic systemic conditions [32]. In the case of TNF-alpha, much of its action is exerted at the paracrine level [33], so it is likely that circulating levels do not reflect actual TNF-alpha secretion, particularly by adipose tissue. Adiponectin levels did not differ significantly between groups, but there was a trend towards lower levels in the at risk group. In any event, in our study reduced adiponectin was not a prominent characteristic of Hispanics at risk for type 2 diabetes.

In our selection of at risk individuals, we combined two separate risk factors, one modifiable and mostly environmental in nature (overweight) and one non-modifiable and mostly genetic in nature (family history). For future studies it will be interesting to explore how much of the observed alterations in markers can be attributed to each risk factor separately, as this will have implications in the development of preventive strategies.

Despite the constellation of other differences in relevant biomarkers between groups, including the presence of some individuals with IGT and IFG, we did not find significantly higher A1c values in the at risk group. This observation brings even more rationale for further studies that clarify the potential role of A1c as a diagnostic test to screen for or to diagnose pre-diabetic states, particularly in this ethnicity and age group [34,35].

## Conclusions

Our results suggest that young adult Hispanics at risk of type 2 diabetes but without clinical or biochemical diabetes already bear considerably high levels of markers reflecting processes that promote the development of CVD. In particular sICAM (endothelial dysfunction), tPA and PAi-1 (hypercoagulability), hsCRP (chronic inflammation) and plasma free fatty acids (adipose dysfunction) may be early markers of these pathogenic processes. Given the growing relevance of cardiovascular and metabolic disease in this population group, these observations constitute a call to early preventive actions focused on young Hispanics.

## Abbreviations

CVD: Cardiovascular disease; sICAM-1: Soluble intercellular adhesion Molecule-1; sVCAM: Soluble vascular adhesion molecule; tPA: Tissue-type plaminogen activator; PAi-1: Plasminogen activator inhibitor-1; hsCRP: High sensitivity C-reactive protein; IL-6: Interleukin 6; JDC: Joslin diabetes center; BMI: Body-mass index; OGTT: Oral glucose tolerance test; AUC: Area under the curve; HOMA-IR: Homeostasis model assessment of insulin resistance; ISI: Insulin sensitivity index; CIR: Corrected insulin response; IFG: Impaired fasting glucose; IGT: Impaired glucose tolerance.

## Competing interests

The authors have no competing interests to disclose.

## Authors’ contributions

COM reviewed the protocol, analyzed the data and wrote the manuscript, LR-O participated in the writing of the protocol and collected research data, ESN participated in the writing of the protocol and critical review of the manuscript, OH participated in the writing of the protocol and critical review of the manuscript, AEC directed and coordinated the project, wrote the protocol, and provided critical review of the manuscript. All authors read and approved the final manuscript.
